# Congenital Microgastria With Esophageal Stricture: An Extremely Rare Foregut Anomaly Necessitating Early Surgical Intervention

**DOI:** 10.14309/crj.0000000000002349

**Published:** 2026-10-01

**Authors:** Arpit Gupta, Ajay Aravind, Anshu Srivastava, Ankur Mandelia, Moinak Sen Sarma

**Affiliations:** 1 Department of Pediatric Gastroenterology, Sanjay Gandhi Post Graduate Institute of Medical Sciences, Lucknow, India; 2 Department of Pediatric Surgery, Sanjay Gandhi Post Graduate Institute of Medical Sciences, Lucknow, India

## CASE REPORT

A 10-month-old boy presented with vomiting of uncurdled milk immediately after feeding since the age of 5 months. The child required multiple hospital admissions for the same and was conservatively managed. At presentation to our center, the infant had severe malnutrition (weight and height <3 SD) and had vomiting with nasal regurgitation of milk when attempted to feed. Clinical possibilities considered were congenital esophageal stricture (CES), peptic stricture, and achalasia cardia. Barium swallow revealed grossly dilated esophagus, a narrowing at the gastroesophageal junction with persistent contrast holdup at 10 minutes, a small stomach, and rapid passage of contrast into the proximal small bowel (Figure 1). This raised suspicion of microgastria. Upper gastrointestinal endoscopy revealed a dilated esophagus with pooling of secretions and a narrowing with pinpoint lumen at the gastroesophageal junction (Figure 2). Computed tomography findings revealed dilated esophagus, no tracheobronchial remnant for CES, and a small, contracted stomach, concordant with congenital microgastria with esophageal stricture (Figure 3). The child underwent gastric augmentation with Hunt-Lawrence pouch (Roux-en-Y) with jejunojejunostomy with division of esophagus and stomach at the level of gastroesophageal junction. At 2 months of follow-up, the child remained asymptomatic with a weight gain of 2 kg.

**Figure 1.**
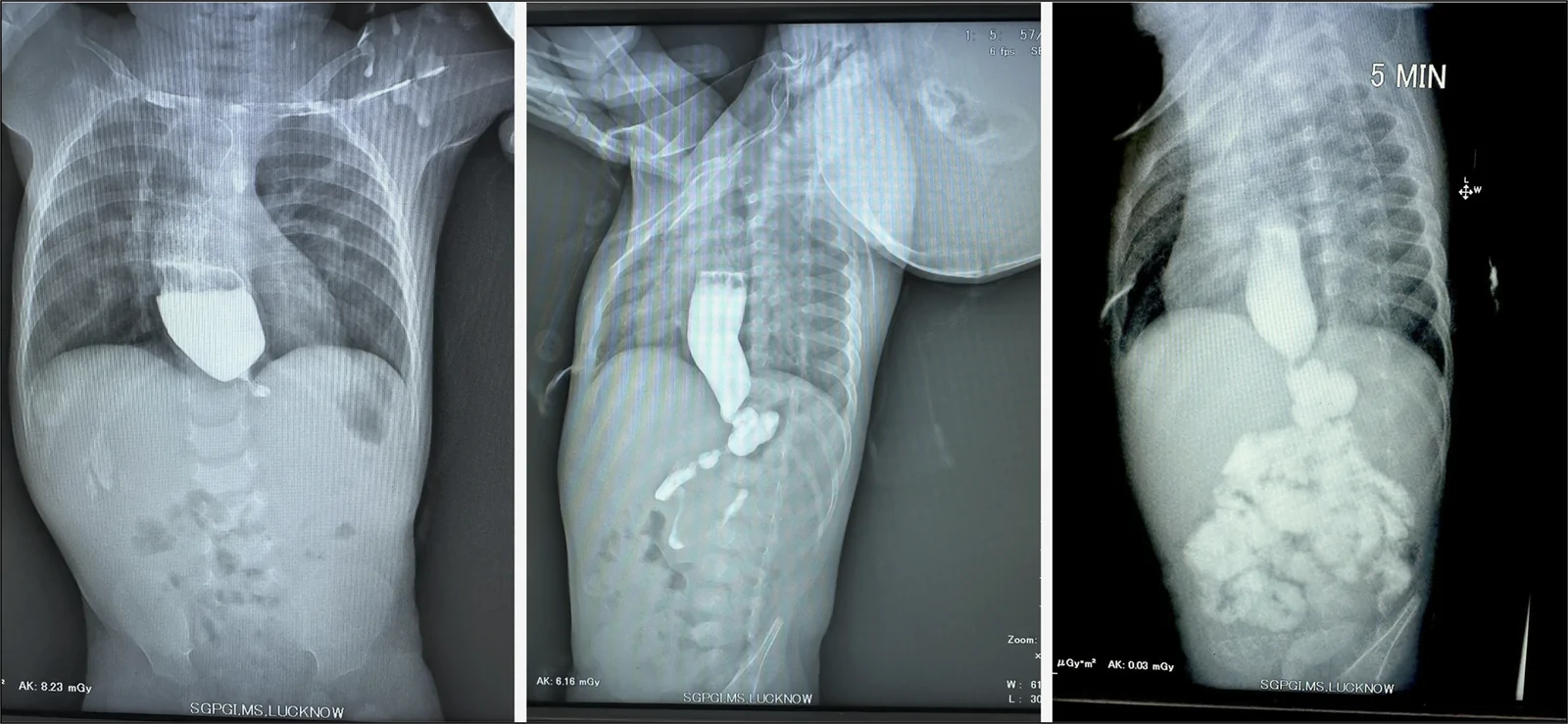
Barium swallow revealed grossly dilated esophagus, a narrowing at the gastroesophageal junction with persistent contrast holdup, a small stomach, and rapid passage of contrast into the small bowel at 5 minutes.

**Figure 2.**
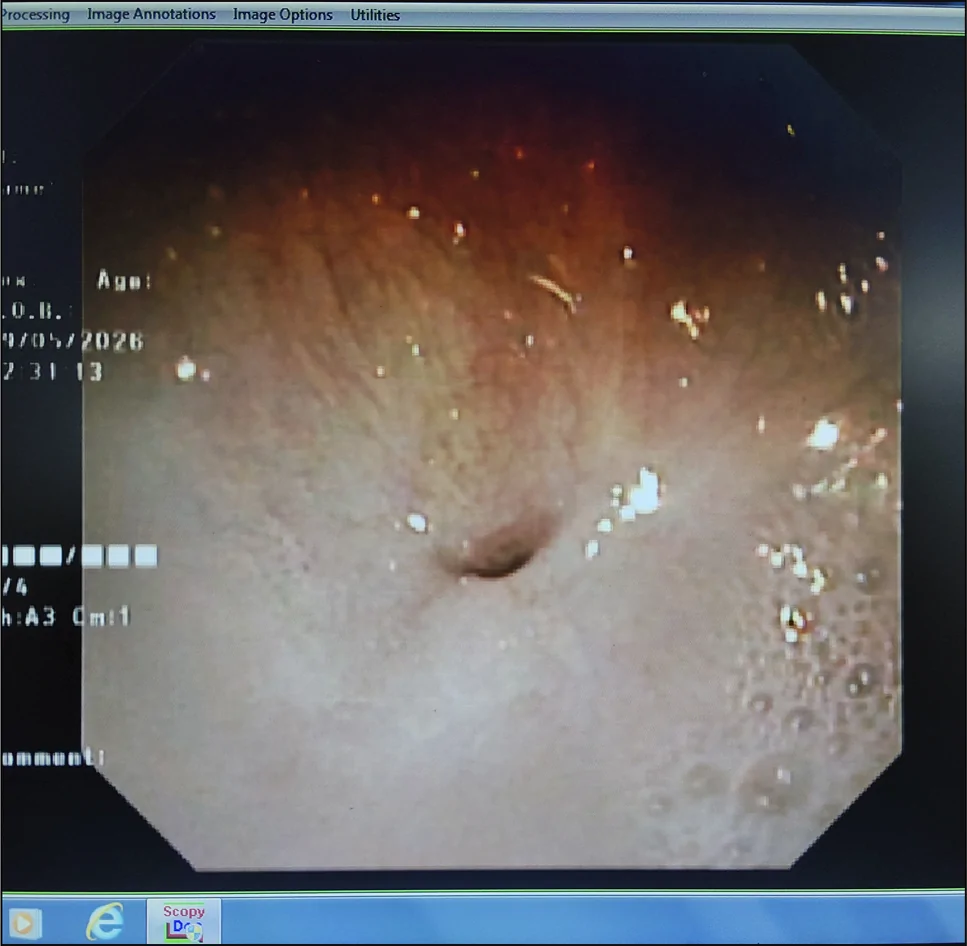
Upper gastrointestinal endoscopy revealed a dilated esophagus with pooling of secretions and a narrowing with pinpoint lumen at the gastroesophageal junction.

**Figure 3.**
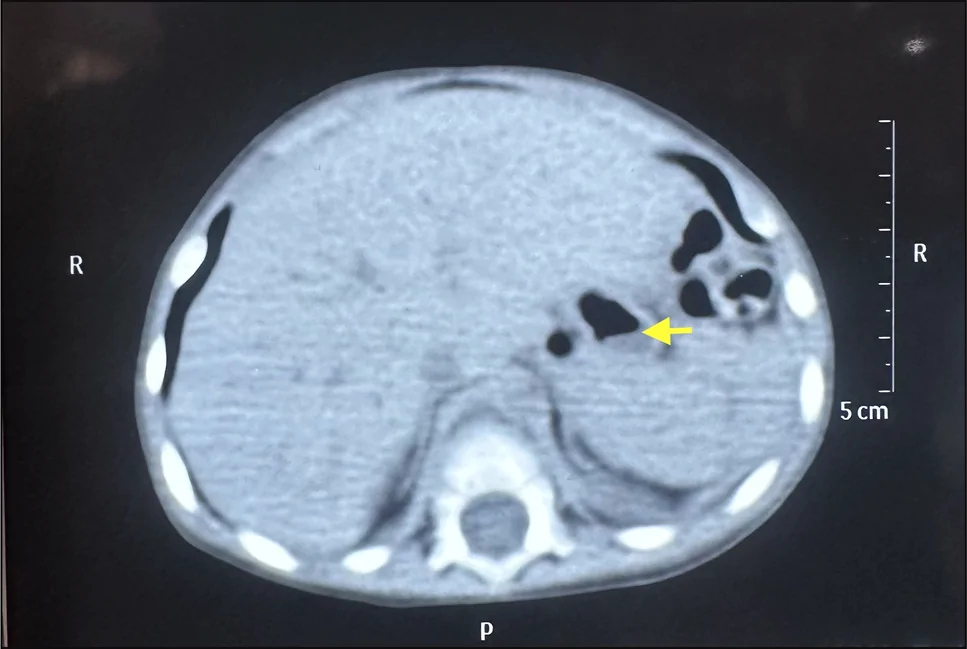
Computed tomography of the abdomen showing small, contracted stomach, concordant with congenital microgastria (yellow arrow).

Congenital microgastria associated with esophageal stenosis is an exceptionally rare foregut anomaly with relatively few published reports.^1,2^ The possible causes of esophageal stricture in congenital microgastria include associated congenital esophageal stenosis and peptic stricture. Early diagnosis and timely surgical intervention can lead to favorable clinical outcome.^3^

## DISCLOSURES

Author contributions: A. Gupta, A. Aravind, A. Srivastava, and A. Mandelia: Substantial contributions in terms of literature review, intellectual input, drafting the article, and provided the final approval of the version to be published. M. Sen Sarma: Substantial contributions in terms of literature review, drafting the article, and provided the final approval of the version to be published. A. Srivastava is the article guarantor.

Financial disclosure: None to report.

Informed consent was obtained for this case report.
